# Mitochondrial and mitochondrial‐independent pathways of myocardial cell death during ischaemia and reperfusion injury

**DOI:** 10.1111/jcmm.15127

**Published:** 2020-03-10

**Authors:** Sean M. Davidson, Adriana Adameová, Lucio Barile, Hector Alejandro Cabrera‐Fuentes, Antigone Lazou, Pasquale Pagliaro, Kåre‐Olav Stensløkken, David Garcia‐Dorado, the EU‐CARDIOPROTECTION COST Action (CA16225)

**Affiliations:** ^1^ The Hatter Cardiovascular Institute University College London London UK; ^2^ Faculty of Pharmacy Comenius University Bratislava Bratislava Slovakia; ^3^ Centre of Experimental Medicine SAS Bratislava Slovakia; ^4^ Laboratory for Cardiovascular Theranostics Cardiocentro Ticino Foundation and Faculty of Biomedical Sciences Università Svizzera Italiana Lugano Switzerland; ^5^ SingHealth Duke‐NUS Cardiovascular Sciences Academic Clinical Programme and Cardiovascular and Metabolic Disorders Program Duke‐National University of Singapore Medical School Singapore Singapore; ^6^ National Heart Research Institute Singapore National Heart Centre Singapore Singapore Singapore; ^7^ Tecnologico de Monterrey Centro de Biotecnologia‐FEMSA Monterrey Nuevo Leon México; ^8^ Institute of Fundamental Medicine and Biology Kazan (Volga Region) Federal University Kazan Russia; ^9^ Institute of Physiology Medical School Justus‐Liebig‐University Giessen Germany; ^10^ School of Biology Aristotle University of Thessaloniki Thessaloniki Greece; ^11^ Department of Biological and Clinical Sciences University of Turin Torino Italy; ^12^ National Institute for Cardiovascular Research Bologna Italy; ^13^ Section of Physiology Department of Molecular Medicine Institute for Basic Medical Sciences University of Oslo Oslo Norway; ^14^ IIS‐Fundación Jiménez Díaz University Hospital Madrid Spain; ^15^ Department of Cardiology Vascular Biology and Metabolism Area Vall d’Hebron University Hospital and Research Institute (VHIR) Barcelona Spain; ^16^ Universitat Autónoma de Barcelona Barcelona Spain

**Keywords:** apoptosis, autophagy, cardiac, cell death, ischaemia, myocardial infarction, necroptosis, necrosis, pyroptosis, reperfusion

## Abstract

Acute myocardial infarction causes lethal injury to cardiomyocytes during both ischaemia and reperfusion (IR). It is important to define the precise mechanisms by which they die in order to develop strategies to protect the heart from IR injury. Necrosis is known to play a major role in myocardial IR injury. There is also evidence for significant myocardial death by other pathways such as apoptosis, although this has been challenged. Mitochondria play a central role in both of these pathways of cell death, as either a causal mechanism is the case of mitochondrial permeability transition leading to necrosis, or as part of the signalling pathway in mitochondrial cytochrome c release and apoptosis. Autophagy may impact this process by removing dysfunctional proteins or even entire mitochondria through a process called mitophagy. More recently, roles for other programmed mechanisms of cell death such as necroptosis and pyroptosis have been described, and inhibitors of these pathways have been shown to be cardioprotective. In this review, we discuss both mitochondrial and mitochondrial‐independent pathways of the major modes of cell death, their role in IR injury and their potential to be targeted as part of a cardioprotective strategy. This article is part of a special Issue entitled ‘Mitochondria as targets of acute cardioprotection’ and emerged as part of the discussions of the European Union (EU)‐CARDIOPROTECTION Cooperation in Science and Technology (COST) Action, CA16225.

## 1 INTRODUCTION

Ischaemic heart disease remains a major cause of morbidity and mortality throughout the world, and is responsible for ~20% of deaths in the European Union in both men and women.^1^ Many of these deaths occur during an acute ischaemic event such as an ST‐elevation myocardial infarction (STEMI). Although fatality rates immediately following acute myocardial infarction have decreased in most countries,^1^ infarct size remains a major determinant of outcome and is strongly associated with all‐cause mortality and hospitalization for heart failure within the following year.^2^ Cardiomyocytes begin to die during exposure to prolonged ischaemia, and while reperfusion is necessary to limit this process, it causes a spike of further cell death that contributes to final infarct size.^3^ Thus, finding ways to limit cardiomyocyte death during ischaemia and reperfusion (IR) has been the focus of extensive studies over the past 30 years.^3^ Myocardial IR is a complex process during which the ability of physiological processes to return the cardiac cells to homeostasis is overwhelmed. A major cause of this is calcium overload which damages cellular components and drains energy (ATP) as ion pumps in the sarcolemma and sarcoplasmic reticulum (SR) are engaged to return cytosolic calcium back to appropriate levels.^4^, ^5^ Mitochondrial calcium overload causes mitochondrial damage and further depletes ATP as it is utilized to maintain mitochondrial membrane potential. In combination with oxidative stress and calcium overload, ATP levels may decrease to a critical level at which the ability of the cardiac cell to remain viable becomes compromised, and the cell undergoes uncontrolled death through a process of oncosis and necrosis, which is described in detail below. However, even before this step, programmed cell death pathways may be activated including apoptosis, necroptosis or pyroptosis. Although ultimately each of these pathways still results in the death of the cell, they can have profoundly different effects on the heart, for example in terms of the activation of an inflammatory response. Furthermore, as many of the pathways appear to overlap or utilize common cellular signalling components, modulation of one pathway may simply result in the cardiomyocyte dying by an alternative pathway. This review aims to provide insight into the different types of cell death which myocardial cells may undergo during IR, with special emphasis on the role of mitochondria in those processes, in order to understand how these processes can be targeted to protect the heart.^6^

It is important to note that the initial description and definition of several of the cell death pathways (apoptosis, pyroptosis, etc) were based on experimental observations in leucocytes, and they may have different manifestations in cardiomyocytes or other non‐inflammatory cell types in the heart. In this review, we focus on cell death pathways occurring in cardiomyocytes. Clearly, mitochondria are central to the function of cardiomyocytes, occupying nearly 40% of the cytosolic volume,^7^ and providing the bulk of the ATP necessary for contraction as well as ion pumps and metabolic processes essential for survival. It is therefore not surprising that mitochondria appear to have a central place in the process of cardiomyocyte death.

## 2 ONCOSIS AND NECROSIS

**Figure 1:**
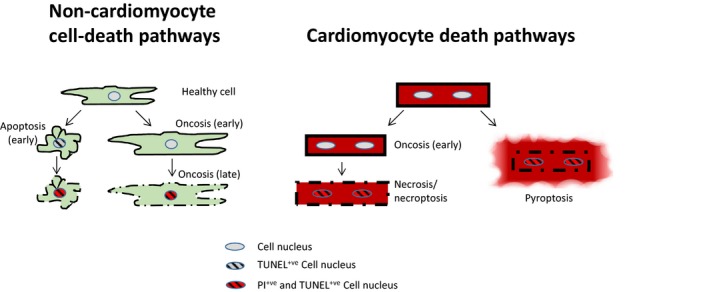
The major pathways of cell death that contribute to myocardial ischaemia and reperfusion injury. During initial oncosis, cells swell—this is reversible but can proceed to necrosis. Non‐cardiomyocytes can die via a processes of apoptosis or necrosis/necroptosis, in addition to other types of cell death described herein. Cardiomyocytes die primarily via a process of necrosis/necroptosis in addition to other cell‐death processes such as pyroptosis, but there is a little evidence for any contribution of apoptosis. Plasma membrane rupture is the terminal event, and this is mediated either by MLKL channels during necroptosis or by GSDMD pores during pyroptosis. Multiple cell‐death pathways can eventuate in plasma membrane permeabilization, as detected by dyes such as propodium iodide (resulting in red nuclei as shown) or trypan blue

During myocardial ischaemia, oxygen is rapidly depleted, causing mitochondrial respiration to cease. Anaerobic metabolism is activated within seconds of flow cessation, but is unable to provide sufficient ATP for maintaining sarcolemmal ion gradients and mitochondrial membrane potential (ΔΨ_m_). ATP is further depleted by the F_0_F_1_ATPase running in reverse, expending ATP in a futile attempt to maintain ΔΨ_m_. Eventually, the sarcolemmal ion pumps fail and the cell swells in a process called ‘oncosis’, which is defined as a pre‐lethal stage following cellular injury.^8^ Shortly afterwards, oncosis leads inevitably to necrosis, defined by the physical, chaotic disruption of the cell membrane. Many techniques for identification of dead (necrotic) cells rely on this permeability of the cell membrane, which allows dyes such as propodium iodide or trypan blue to enter and stain the cell (Figure 1). However, it is important to note that membrane permeabilization, and hence necrosis, can occur secondarily to cell death by any mechanism, including the later phase of apoptosis. Likewise, it should also be noted that various cell death modalities with the features of ruptured plasma membrane can occur in parallel. These facts can make it difficult to ascertain the precise cause of myocardial cell death, particularly when examining a single time‐point.

Mitochondria play a critical role in the process of myocardial IR injury. In particular, upon reperfusion, when the supply of oxygen to the cardiac cells is re‐introduced and mitochondrial respiration recommences. The mitochondrial substrate succinate, having accumulated during ischaemia, provides a powerful source of electrons which produce oxygen radicals by reverse electron transport via complex I, resulting in oxidative stress (excess reactive oxygen species or ROS).^9^ The rapid replenishment of oxygen and ATP to the cells is a double‐edged sword. ATP is necessary to restore ionic homeostasis but it also reactivates the sarcoplasmic reticulum ATPASE 2A (SERCA2A) allowing it to pump Ca^2+^ back into the SR. However, hyperactivation of the SR Ca^2+^ release channel, RyR2, results in rapid cycles of SR Ca^2+^ uptake and spontaneous SR Ca^2+^ release. The restoration of ATP levels while cytosolic Ca^2+^ overload is still current also leads to hypercontraction of cardiomyocytes, which can be detected during the first few minutes of reperfusion by the appearance of ‘contraction band necrosis’ in haematoxylin and eosin‐stained myocardial histological sections.^10^ The excessive SR Ca^2+^ release contributes to mitochondrial Ca^2+^ uptake via the mitochondrial calcium uniporter (MCU), leading to mitochondrial Ca^2+^ overload and opening of the mitochondrial permeability transition pore (MPTP).^11^ With the MPTP open, the mitochondria are no longer able to maintain ΔΨ_m_, and shortly afterwards, ATP stores are depleted, ion pumps cease functioning, and the cells die through a process of oncosis and necrosis.

It may be possible to target necrosis during the early stages of infarction in order to protect the heart from IR injury. Mice lacking either the MPTP or the MCU have smaller infarct sizes following IR.^12^, ^13^, ^14^ Cardioprotection against IR injury can also be achieved experimentally by activation of the MAPK/ERK1/2 or PI3K/AKT signalling pathways.^15^, ^16^ Activation of the reperfusion injury salvage kinase (RISK) pathway protects the heart by delaying opening of the MPTP.^17^ At least in the isolated, perfused heart, blocking hypercontraction by lowering pH or administering inhibitors of the contractile machinery reduces infarct size, as does blocking of reverse electron flow by providing malonate.^18^, ^19^, ^20^ However, as will be discussed later, it cannot be ruled out that such previously reported MPTP opening associated with necrosis^17^ can be also a mechanism of other necrosis‐like cell death modes, which have been identified more recently.^13^

## 3 APOPTOSIS

Apoptosis is a form of cell death that can be distinguished microscopically from oncosis by cell shrinkage, chromatin condensation and distinctive blebbing (budding) of the plasma membrane. Apoptosis can occur via intrinsic or extrinsic mechanisms but both result in mitochondrial outer membrane permeabilization (MOMP), mitochondrial cytochrome c release, caspase activation, DNA fragmentation and cell blebbing.^21^

Early studies detected evidence of apoptosis along with necrotic cell death following myocardial IR.^22^ However, the relative contribution of apoptosis to the extent of cardiac damage is still debated due to the large differences in its magnitude as reported by different investigators. DNA laddering, one of the hallmarks of apoptosis, was not detected in myocardium subjected to ischaemia alone, but was only observed after reperfusion, suggesting that the apoptotic component of cell death in the myocardium is triggered at the time of reperfusion and does not manifest during the ischaemic period.^23^, ^24^ In contrast, other studies have shown that apoptosis begins either after prolonged myocardial ischaemia without reperfusion or after a brief period of ischaemia followed by reperfusion.^25^, ^26^ Detection of pro‐apoptotic factors and caspase activation during ischaemia in the absence of DNA fragmentation followed by a more massive increase during reperfusion indicates that the apoptotic cascade is initiated during ischaemia, but is fully executed during reperfusion.^27^, ^28^ More supportive evidence for the acceleration of apoptosis during reperfusion comes from studies showing a reduction in infarct size using inhibitors of pro‐apoptotic mediators at early reperfusion.^29^, ^30^ Studies in humans have also demonstrated the detection of apoptotic cardiomyocytes in the border zone of the infarcted myocardium within hours to days of infarction.^31^

In contrast to the above, other studies have argued against the significant role of apoptosis in IR‐induced cell death, based on the fact that there is minimal expression of most proteins required for the apoptotic program in adult cardiomyocytes.^32^, ^33^ In addition, using cardiac‐specific knockout mice, it was conclusively shown that the executioner caspase‐3 and caspase‐7 do not significantly contribute to the acute effects of myocardial IR injury.^34^ Even forced overexpression of caspase in cardiomyocytes is not able to trigger a full apoptotic response in cardiomyocytes during IR, although it does result in increased infarct sizes.^35^ This raises the possibility that the previous observations of apoptosis in the heart are likely to have been due to apoptosis of non‐cardiomyocytes.^21^, ^32^ Notably, as non‐cardiomyocytes significantly outnumber cardiomyocytes in the heart, their apoptosis could easily account for the observed DNA laddering. Similarly, TUNEL staining is weak evidence for cardiomyocyte apoptosis because ultrastructural studies have shown that TUNEL staining is present only in cardiomyocytes that have already died by necrosis.^36^ Of note, apoptosis of endothelial cells and leucocytes would also be expected to indirectly affect cardiomyocyte cell survival and cardiac performance.^32^ Thus, anti‐apoptotic strategies may still be cardioprotective.^21^ For example, a recently developed peptide targeting the FAS‐dependent apoptotic signal during IR injury decreased infarct size in mice, even when administration was delayed 30 minutes into reperfusion.^37^

Mitochondria play an important role in the execution of apoptosis. These organelles are the major contributors of ROS and the major target for ROS‐induced damage. The mitochondrial apoptosis pathway is triggered by mitochondrial swelling and outer mitochondrial membrane rupture, thus favouring the release of pro‐apoptotic factors such as cytochrome c and SMAC/Diablo from the intermembrane space into the cytosol.^38^ Oxidative stress and Ca^2+^ overload leading to MPTP opening may contribute to apoptosis by increasing MOMP and cytochrome c release.

Another important mechanism for mitochondrial quality control is mitochondrial fusion and fission. Mitochondrial fission is considered to be the prerequisite for the occurrence of mitophagy and several studies revealed the causal relationship between mitochondrial fission and the induction of apoptosis.^39^, ^40^ In this respect, it has been demonstrated that increased mitochondrial fission in the ischaemic heart contributes to apoptosis induction and infarct generation, while inhibiting mitochondrial fission reduces myocardial injury and improves cardiac function following myocardial infarction.^41^

## 4 AUTOPHAGY

Mitochondrial damage negatively affects cardiomyocyte function via disruption of oxidative phosphorylation, Ca^2+^ dyshomeostasis, increased oxidative stress and incomplete digestion of mitochondrial DNA. These events play a role in triggering the formation of inflammasomes within cells.^42^ Thus, a process that contributes to the elimination of toxic mitochondrial contents and mitochondrial quality control is crucial to the maintenance overall cardiomyocyte health. Mechanisms of mitochondrial degradation include mitochondrial autophagy, also known as mitophagy, and micromitophagy, whereby lysosomes directly fuse with and degrade mitochondria.^43^ A well‐established mechanism of mitophagy in cardiomyocytes requires the accumulation of mitochondrial PTEN‐induced putative kinase‐1 (PINK1) at depolarized mitochondrial outer membranes, which promotes translocation of the E3 ubiquitin ligase Parkin from the cytosol to the damaged mitochondria.^44^ PINK1‐mediated phosphorylation of Parkin induces a conformational change in the active form which ubiquitinates mitochondrial surface proteins such as the voltage‐dependent anion channel‐1 (VDAC1) and Mitofusin 1, 2 (MFN1, 2), leading to mitochondrial autophagic removal.^45^, ^46^ Another E3 ubiquitin ligase, TNF‐receptor‐associated factor 2 (TRAF2), has also been shown to promote the removal of ubiquitin‐tagged and damaged mitochondria during IR injury, but in a Parkin‐independent manner.^47^ Two further Parkin‐independent mechanisms of mitophagy involve mitochondrial outer membrane proteins: the mitochondrial pro‐apoptotic BH3 domain‐only protein BNIP3 and FUNDC1.^48^ BNIP3 is up‐regulated in the myocardium during hypoxia^49^ and promotes mitophagy that does not require ubiquitination. Under hypoxic conditions, FUNDC1 is dephosphorylated by phosphatases (eg PGAM5) at Serine 13, which induces protein interaction with microtubule‐associated proteins 1A/1B light chain 3 (LC3), thereby enhancing mitophagy.^50^, ^51^

Mitophagy can be considered a beneficial cellular process that enhances cell viability following stressful stimuli by eliminating dysfunctional mitochondria. Mitophagy is therefore essential for cardiomyocyte survival.^52^ Accumulated evidence suggests that IR causes an imbalance in the mitophagy process, and one could easily imagine how this would allow dysfunctional mitochondria to accumulate in the cell, causing further cytotoxic damage and potentially leading to cell death. However, it remains controversial whether it is excessive mitophagy or large‐scale accumulation of autophagosomes that is the main mechanism underlying ‘autophagic cell death’.^40^, ^53^, ^54^ The emerging consensus is that cellular insults induce changes consistent with autophagosome formation and the initiation of autophagy in cardiac cells, and that these processes can lead to cell death.

The results of early studies of the role of autophagy in IR‐induced cardiomyocyte death were somewhat contradictory, indicating that autophagy could be cyto‐protective, but could also direct cells towards apoptosis.^55^ These observations are in contrast with the notion that autophagy may trigger cell death in a caspase‐independent way as assessed in vitro^56^ and in vivo*,* 57 where it has been shown that impairing the expression of ATG genes leads to reduction of cell death. However, rigorous kinetic analyses are required to establish whether autophagic cell death is independent from apoptotic or necrotic processes and whether it represents a step through which these processes culminate with cell disruption.^53^ Evidence from Sadoshima's group suggested that autophagy is beneficial during ischaemia but harmful during reperfusion.^58^ However, more recently, the balance of evidence favours a beneficial role for autophagy in the heart under most conditions.^59^

## 5 NECROPTOSIS

**Figure 2:**
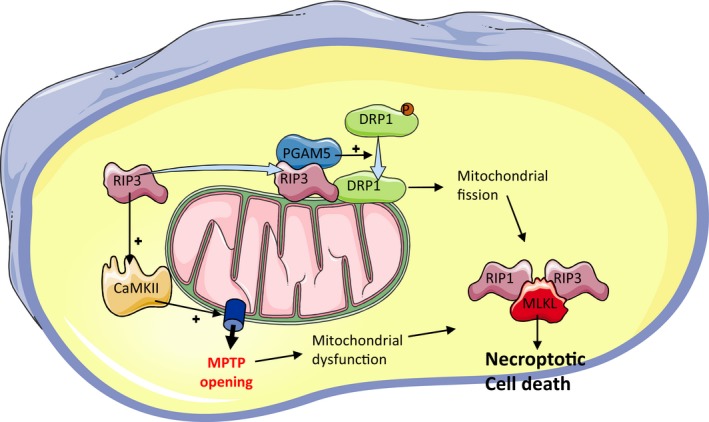
Possible mitochondrial pathways of necroptosis. Ischaemia and reperfusion causes an increase in levels or phosphorylation and activation of RIP1 and RIP3 (receptor‐interacting serine/threonine‐protein kinase 1 and 3), MLKL (mixed‐lineage kinase domain‐like pseudokinase) and PGAM5 (phosphoglycerate mutase family member 5). RIP3 translocates to the mitochondria and interacts with PGAM5 and DRP1. PGAM5 dephosphorylates and activates DRP1 leading to mitochondrial fission and dysfunction. Additionally, RIP3‐mediated activation of CaMKII (Ca^2+^‐calmodulin‐dependent protein kinase) may trigger necroptosis via the MPTP. Mitochondrial dysfunction leads ultimately the formation of the necroptosome consisting of activated RIP1, RIP3 and MLKL, and necroptotic cell death ensues.

Necroptosis, a regulated mode of cell death with a necrotic appearance, has been identified in various cardiac pathologies, including myocardial IR (reviewed in^60^, ^61^). The precise cytotoxic mechanisms of necroptosis are not fully understood; however, the activation of RIP1 and RIP3 (receptor‐interacting serine/threonine‐protein kinase 1 and 3) is essential for necroptotic cell membrane rupture‐inducing events that occur as a consequence of the relocalization to the plasma membrane of phosphorylated mixed‐lineage kinase domain‐like pseudokinase (MLKL) (Figure 2).^62^ Accordingly, the majority of studies of both acute^63^, ^64^, ^65^, ^66^, ^67^ and chronic myocardial IR injury progressing into heart failure^68^, ^69^, ^70^ indicate that membrane‐associated RIP1‐RIP3‐MLKL axis is a key player in necroptotic damage leading to worsening of heart function and adverse cardiac remodelling. However, only a very limited number of experiments have investigated the importance of mitochondria in this process. By using a pharmacological inhibitor of RIP1, necrostatin‐1 (Nec‐1), which reduced infarct size in wild‐type but not in CypD^−/−^ mice, it has been suggested that cyclophilin D, an important regulatory component of the MPTP, might be involved in necroptosis signalling^66^ due to promotion of MPTP opening.^67^ RIP3‐mediated activation of CaMKII (Ca^2+^‐calmodulin‐dependent protein kinase), serving as an upstream regulator of the MPTP, is likely to trigger this critical event in IR‐induced necro(pto)sis.^71^

Necroptosis has been seen to occur in both H9c2 cardiomyoblasts subjected to hypoxia/reoxygenation (HR) and in vivo rat hearts subject to IR, as evidenced by increased levels of RIP1, RIP3 and MLKL.^72^ An increase was also seen in the mitochondrial membrane protein PGAM5 (phosphoglycerate mutase family member 5), an atypical Ser/Thr phosphatase that dephosphorylates and activates the mitochondrial fission protein DRP1.^72^ Furthermore, PGAM5 knockdown suppressed the generation of ROS and preserved ΔΨ_m_, implicating mitochondria in the process of necroptosis.^72^ In a similar model of H9c2 subjected to HR, siRNA targeting RIP3 was shown to prevent mitochondrial fragmentation and necroptosis, by preventing activation of DRP1, again suggesting a link between necroptosis and mitochondria.^73^

Subcellular analyses have further revealed that RIP3 co‐localizes with mitochondria^69^ or translocates to mitochondria during HR and interacts with DRP1, but not with RIP1 and MLKL.^73^ This provides evidence for necroptosis execution being independent of the canonical pathways these proteins are involved in.^71^ These findings suggest that RIP3‐PGAM5‐DRP1 cause mitochondrial dysfunction that promotes necroptosis. However, in contrast, Lu et al found that PGAM5 ablation was deleterious because it increased infarct size and exacerbated necroptosis.^74^ Promotion of mitophagy, which can prevent necroptosis by clearing ROS‐producing unhealthy mitochondria,^75^ can underlie such a protective regulatory function of this protein phosphatase in necroptosis‐associated conditions.

From the above discussion, it is evident that the extent of mitochondrial participation in necroptosis in cardiomyocytes or other cell types is not clear. It has been suggested that the contribution of mitochondria to necroptosis may be secondary or context‐dependent.^76^ For instance, MLKL, the ultimate effector of necroptosis, is unlikely to directly assemble at mitochondrial membranes, as shown for the much higher efficiency of the MLKL N‐terminal domain to permeabilize liposomes with a composition resembling the plasma membrane, in comparison with cardiolipin‐rich liposomes.^77^ Nonetheless, at early stages of SMAC‐mimetic induced necroptosis in various cancer cell types, a disruption of mitochondrial membrane potential is observed that presumably depends on BAK‐BAX activity.^78^ In accordance with this, the pro‐apoptotic protein PUMA could act as an amplifier of necroptosis by exposing mitochondrial DNA to the cytosolic sensors, which further stimulates the necrosome formation.^79^ Moreover, at the inner mitochondrial membrane, MPTP response could be an important mediator of this kind of cell death, as either cyclosporine A treatment or cyclophilin D deficiency confer resistance to necroptosis prompted by TNFα exposure of endothelial cells.^80^ In summary, therefore, while specific mitochondrial mechanisms in the membrane canonical pathway of necroptosis cannot be ruled out, the details remain to be elucidated.

## 6 PYROPTOSIS

Pyroptosis, meaning ‘fire’ and ‘falling’, is a pro‐inflammatory cell‐death program occurring after cytosolic receptor‐mediated recognition of pathogen‐associated molecular patterns (PAMPs), or host‐derived, danger signals such as damage‐associated molecular patterns (DAMPs). Some well‐characterized DAMPs include glucose‐regulated proteins (GRPs), high‐mobility group box 1 (HMGB‐1), IL‐1β, S100 family proteins and some heat shock proteins (HSPs). Interaction of these proteins with cellular pattern recognition receptors can lead to the assembly of the intracellular NLRP3 inflammasome complex. The assembled NLRP3 inflammasome elicits auto‐proteolytic cleavage and activation of caspase‐1, which mediates the cleavage/maturation of the pro‐inflammatory cytokines, pro‐IL‐18 and pro‐IL‐1β, and cleaves/activates gasdermin‐D (GSDMD), releasing its N‐terminal fragment.^81^ Activated GSDMD‐N, as well as active caspase‐1, may induce the formation of membrane pores thus inducing cell lysis, called pyroptotic cell death.^82^, ^83^, ^84^ The subsequent immune response triggered by DAMPs can lead to various forms of cell death, including apoptosis, pyroptosis, necrosis and necroptosis. Of note, bidirectional crosstalk between pyroptotic and apoptotic cell death mechanisms has been described: caspase‐1 can cleave and activate caspase‐3 and caspase‐7 to start apoptosis, which in turn can cleave and inactivate GSDMD to limit pyroptosis.^85^ Likewise, a crosstalk between pyroptosis and necroptosis being associated with RIP3 and MLKL activation has also been suggested.^86^

The DAMPs released after cell death can induce further injury, thus causing a *vicious cycle* that expands the region of ischaemic damage. Evidence for a role of inflammasome/pyroptosis in acute myocardial IR injury comes from several studies.^87^ In 2001, preceding the notion of the inflammasome, it was reported that the secretion of IL‐1β and IL‐18 was increased in an IR model of isolated human atrial myocardium.^88^ In 2003, targeted deletion of caspase‐1 was shown to reduce early mortality and left ventricular dilation following cardiac infarction in mice, thus supporting a link between inflammasome activation, apoptosis and cardiovascular diseases.^89^ In 2011, Kawaguchi et al^90^ reported an essential role for cardiac fibroblasts inflammasome activation in myocardial IR injury. Simultaneously, Mezzaroma et al,^91^ using a gene silencing NLRP3 model, suggested that NLRP3 overexpression occurs not only in cardiac fibroblasts, but also in infiltrating cells and, importantly, in cardiomyocytes of the border zone of the infarct area. In 2013, Sandanger et al reported a reduced infarct size in Nlrp3^−/−^ mice in an ex vivo Langendorff perfused heart model of IR.^92^ Also using isolated hearts, it was confirmed that pre‐treatment with the NLRP3 inhibitor, INF4E, reduced infarct size and improved ventricular developed pressure after IR.^93^ These findings were confirmed by Luo et al in a type 2 diabetes rat model.^94^

The mechanisms of myocardial IR damage by DAMPs may involve binding to receptor for AGE (advanced glycation end products, RAGE) or to TLR4 (toll‐like receptor 4), thus activating NFκB and exacerbating myocardial damage. Indeed, among pro‐inflammatory genes, NFκB promotes the transcription of components of the NLRP3 inflammasome in cardiac cells.^95^ Therefore, the NLRP3 inflammasome may be considered a sensor that links myocardial damage to inflammation, thereby contributing to the progression of the wavefront of IR injury.^96^, ^97^ After activation, the NLRP3 inflammasome promotes cardiomyocyte death and infarct size progression in the first hours of reperfusion likely through pyroptosis and then through production of IL‐1β and inflammation.^91^, ^96^

Despite the fact that IR‐damaged myocardium releases a combination of priming and triggering factors of the NLRP3 inflammasome, it has been proposed that the NLRP3 inflammasome in the heart is not sufficient to respond to a trigger signal in the absence of a priming.^98^ Actually, after IR, the size of the infarct is found to increase more in the presence of an active NLRP3,^93^, ^96^, ^99^ especially if metabolic syndrome has primed the inflammasome.^99^

Although little is known about the role of mitochondria in pyroptosis occurring in the myocardium specifically, there is evidence for mitochondrial involvement in other cell types. In fact, mitochondrial dysfunction, leading to oxidative stress and mitochondrial DNA (mtDNA) release, is emerging as a key mechanism in triggering NLRP3 assembly and activation in several conditions and indeed has been proposed as the universal trigger for NLRP3 activation.^100^, ^101^ Mitochondrial ROS induce NLRP3‐dependent lysosomal damage and further inflammasome activation.^95^, ^102^ ROS favour the mitochondrial localization of NLRP3 and ASC for further NLRP3 activation. VDAC1‐induced ROS formation and BAX/BAK activation can also trigger NLRP3 activation. Calcium overload and calcium entry into mitochondria may favour MPTP opening thus generating ROS, which promote the deubiquitylation of NLRP3 and inflammasome activation, thus facilitating mtDNA release to further activate NLRP3.^103^ TLR4‐IRF1 mediates transcriptional up‐regulation of UMP‐CMPK2 (uridine/cytidine monophosphate kinase‐2), which favours mtDNA synthesis and the formation of oxidized short strands of mtDNA that exit the mitochondria.^104^ Also, exposed mitochondrial cardiolipin can tether NLRP3 to the mitochondria for activation in a ROS‐dependent and ROS‐independent fashion.^105^ Released cardiolipin in combination with ineffective mitophagy lead to NLRP3‐mediated activation of caspase‐1 and subsequent production of IL‐1.^105^ Loss of BCL‐XL and MCL‐1 activity can activate BAX/BAK, which may favour NLRP3 activation, via downstream effectors (activated caspase‐3 and caspase‐7), in a K^+^ efflux‐dependent way. Furthermore, the serine threonine kinase NIMA‐related kinase 7 (NEK7) is a promoter of NLRP3 inflammasome assembly downstream of ROS and K^+^ efflux.^106^, ^107^ In fact, the catalytic domain of NEK7 interacts with the NACHT/LRR domain to favour NLRP3 inflammasome activation.^106^, ^107^ Intriguingly, this interaction may be disrupted by cytochrome c, an intrinsic/mitochondrial regulator of apoptosis, to limit pyroptotic cell death, in a sort of yin yang, bidirectional process between apoptosis and pyroptosis.^85^, ^107^ Finally, it has also been proposed that mitophagy may dampen NLRP3 activation by removing injured mitochondria. Actually, NLRP3 activity and metabolic disease progression have been associated with low levels of mitochondrial mitofusin proteins and elevated levels of DRP1, especially in hyperglycaemic conditions.^108^, ^109^ Yet, deletion of DRP1 may lead to an increase in NLRP3‐dependent caspase‐1 activation and IL‐1 secretion.^110^

Recent evidence has shown that autophagy is necessary to reduce myocardial damage after acute myocardial infarction, thus confirming that the autophagic process can limit the activation of the NLRP3 inflammasome by removing damaged mitochondria and that impaired mitophagy may contribute to adverse cardiac remodelling in myocardial infarction.^111^, ^112^

Arguing against an important role for NLRP3 inflammasome in cardiac injury is a work of Sandanger et al who reported that NLRP3 inflammasome activation is cardioprotective during myocardial IR.^113^ Moreover, Hermansson et al found that NLRP3 levels were low in human ischaemic myocardial tissue compared with non‐ischaemic control cardiac tissue.^114^ However, gene analysis showed mutations in NLRP3 in human cardiac tissues from ischaemic patients, but not in those from non‐ischaemic controls; the authors suggested that genetic defects in the inflammasome and related proteins may represent a background for promoting ischaemic cardiac disease. Yet, Zuurbier et al,^115^ testing the hypothesis that NLRP3 inflammasome, plays a role either in preconditioning (IPC) or in acute IR injury, observed that NLRP3 deletion did not affect cell death, but exacerbated IR‐induced mechanical dysfunction. Moreover, NLRP3 deletion abrogated the protective effects of IPC against IR damage. The authors proposed that the observed effects are due to an altered IL‐6/STAT3 dependent mechanism. Therefore, it is possible that some concomitant modifications of protective pathways may explain discordant results.^116^

## 7 OTHER TYPES OF CELL DEATH

Although the involvement of mitochondria in extrinsic and intrinsic apoptotic pathways is undeniable, and their role in necroptosis and pyroptosis is being elucidated, the degree to which mitochondria are involved in other, more newly recognized, forms of cell death has just begun to be disentangled. In this respect, ‘parthanatos’ entails cell death characterized by excessive activation of poly(ADP‐ribose) polymerase‐1 (PARP‐1).^117^ PARP‐1 transfers ADP‐ribose to apoptosis‐inducing factor (AIF), which in turn translocates from mitochondria to the nucleus where it triggers large‐scale DNA fragmentation and thus precipitates cell death.^118^, ^119^ Hence, mitochondria are central to PARP‐1‐mediated cell death. Genetic disruption of this pathway protects mice against IR injury.^120^

‘Ferroptosis’ is a form of programmed cell death dependent on iron. Ferroptosis seems to converge on the BCL2‐family protein member BID, promoting its translocation to the mitochondria where it causes profound mitochondrial fragmentation and dysfunction.^121^ This suggests a major role for mitochondria during ferroptotic death. However, integrity of the anaplerotic mitochondrial glutaminolysis and the tricarboxylic acid cycle is indispensable for ferroptosis produced by erastin or cysteine starvation but not by glutathione peroxidase 4 deficiency.^122^ Besides, coenzyme Q, an essential electron carrier of the mitochondrial electron transport chain, ought to be able to suppress ferroptosis caused by glutathione peroxidase 4 degradation by an as‐yet‐unknown mechanism, as the hydrophilic coenzyme Q analogue, idebenone, counteracts FIN56‐induced death.^123^ Furthermore, the outer membrane protein CDGSH iron sulphur domain 1 (CISD1) negatively regulates mitochondrial iron accumulation, lipid peroxidation and erastin‐induced ferroptosis.^124^

## 8 TRANSLATIONAL PERSPECTIVES

Although chemical inhibitors of specific cell death pathways are very effective in limiting infarct size after IR in the experimental setting, few studies have investigated these drugs in patients experiencing cardiac IR.^3^, ^6^, ^16^ The paucity of studies is partly because available cell death inhibitors may not be suitable for use in patients, due to insufficient specificity or incompletely determined pharmacokinetics or pharmacodynamics. One exception is the CIRCUS trial which investigated whether CsA would prevent MPTP and mitochondrial‐mediated necrosis in STEMI patients; however, this unfortunately failed to provide evidence of benefit.^125^ There are many potential reasons for this, including the high prevalence of co‐morbidities and the pharmacological background of patients, which may modify cell death pathways. This is discussed further in the accompanying review in this series, ‘Translational issues and mitoprotection’. One further explanation may be that cardiomyocytes that escape early MPTP‐mediated death can go on to die via other pathways.^126^ If so, this may mean that such a single‐target approach will always be destined to fail, and an approach targeting multiple cell death and/or cell survival pathways may be necessary.^4^

## 9 CONCLUSIONS

An important limitation in all investigations of cell death pathways is the methodology used to evaluate cell death in cardiomyocytes (Table 1). TUNEL staining can be misleading as it alone does not distinguish between cells undergoing apoptosis, necrosis or DNA repair.^62^ Caspase activity can be measured but is also not specific to apoptosis as caspases can be involved in other pathways of cell death. Cell‐based assays for more recently described forms of cell death such as pyroptosis and necroptosis have not yet been developed and many used assays are rather nonspecific being limited to the detection of cell damage due to membrane rupture and impairment of cell metabolic activity. Distinguishing between different forms of cell death is further complicated by the potential overlap between them, and sharing of common signalling components. In this regard, recently published guidelines offer useful advice to the use of different assays of cell death.^62^ However, complicating this analysis is the fact that different types of cell can exhibit different characteristics during cell death, and cardiomyocytes may exhibit some particular differences to other cell types. Using observations taken from immune cells to interpret the response of cardiomyocytes to injury may not always lead to the correct interpretation and must be performed with care.

**Table 1:** The key characteristics of the main cell death processes discussed in this review, and their manifestation in cardiomyocytes (if known)

Mode of cell death	Key characteristics	Characteristics known to occur in cardiomyocytes
Necrosis	Plasma membrane permeability; MPTP opening^13^	MPTP opening^13^; Plasma membrane permeability; Hypercontraction/contraction band necrosis^10^
Apoptosis	Mitochondrial outer membrane permeabilization (MOMP), Mitochondrial cytochrome c release, caspase activation, DNA fragmentation cell shrinkage, Chromatin condensation, Plasma membrane blebbing^21^	Controversial; Mitochondrial fission^41^
Autophagy	LC3‐II; Pink1/Parkin accumulation at mitochondria^44^; BNIP3 up‐regulation^49^; FUNDC1‐ser13 dephosphorylation^51^	Similar to the process in other cell types^58^, ^59^
Necroptosis	Recruitment of cytosolic adaptor proteins to complex I^62^; Plasma membrane permeability; relocalization of pMLKL to the plasma membrane^62^; RIP3 activation	Similar to the process in other cell types 62; RIP3‐mediated activation of CaMKII^71^
Pyroptosis	NLRP3 inflammasome formation; Cleavage of GSDMD, caspase‐1, pro‐IL‐18 and pro‐IL‐1β^82^, ^83^, ^84^	Believed to be similar to the process in other cell types^87^, ^91^
Parthanatos	Activation of poly(ADP‐ribose) polymerase‐1 (PARP‐1)^117^	Believed to be similar to the process in other cell types
Ferroptosis	Bid translocation to the mitochondria^121^; Loss of glutathione peroxidase 4^122^	Believed to be similar to the process in other cell types

Note

As some processes can overlap, not all features are necessarily diagnostic of the type of cell death and may depend on the time‐point being examined (eg: most forms of cell death will ultimately result in plasma membrane permeability).

In summary, mitochondria seem to be a convergent point between various regulated cell death processes. In some cases, such as MPTP‐mediated necrosis, apoptosis and parthanatos, their participation is clear, whereas in other cases, such as necroptosis, the extent of their involvement might be context‐dependent. In either case, increasing evidence points to a crosstalk between the diverse pathways of death, with mitochondria likely to be a central node of all such pathways. They are therefore a key target for maintaining the health of cardiomyocytes during IR and thereby protecting the heart from injury.

## CONFLICT OF INTEREST

The authors confirm that there are no conflicts of interest.

## AUTHOR CONTRIBUTIONS

Each author drafted and critically revised a section the paper; SD critically revised the entire manuscript; and all authors approved the entire submitted and final versions.
